# Impact of Aging on the Regenerative Properties of Bone Marrow-, Muscle-, and Adipose-Derived Mesenchymal Stem/Stromal Cells

**DOI:** 10.1371/journal.pone.0115963

**Published:** 2014-12-26

**Authors:** Olivia S. Beane, Vera C. Fonseca, Leroy L. Cooper, Gideon Koren, Eric M. Darling

**Affiliations:** 1 Center for Biomedical Engineering, Brown University, Providence, Rhode Island, United States of America; 2 Department of Molecular Pharmacology, Physiology, and Biotechnology, Brown University, Providence, Rhode Island, United States of America; 3 Cardiovascular Research Center, Rhode Island Hospital, Alpert Medical School of Brown University, Providence, Rhode Island, United States of America; 4 Department of Orthopaedics, Brown University, Providence, Rhode Island, United States of America; 5 School of Engineering, Brown University, Providence, Rhode Island, United States of America; Georgia Regents University, United States of America

## Abstract

Mesenchymal stem/stromal cells (MSCs) are promising cell sources for regenerative therapies due to their multipotency and ready availability, but their application can be complicated by patient-specific factors like age or illness. MSCs have been investigated for the treatment of many musculoskeletal disorders, including osteoarthritis and osteoporosis. Due to the prevalence of these diseases in older populations, researchers have studied how aging affects MSC properties and have found that proliferation and differentiation potential are impaired. However, these effects have never been compared among MSCs isolated from multiple tissue sources in the same, healthy donor. Revealing differences in how MSCs are affected by age could help identify an optimal cell source for musculoskeletal therapies targeting older patients. MSCs were isolated from young and old rabbit bone marrow, muscle, and adipose tissue. Cell yield and viability were quantified after isolation procedures, and expansion properties were assessed using assays for proliferation, senescence, and colony formation. Multipotency was also examined using lineage-specific stains and spectrophotometry of metabolites. Results were compared between age groups and among MSC sources. Results showed that MSCs are differentially influenced by aging, with bone marrow-derived stem cells having impaired proliferation, senescence, and chondrogenic response, whereas muscle-derived stem cells and adipose-derived stem cells exhibited no negative effects. While age reduced overall cell yield and adipogenic potential of all MSC populations, osteogenesis and clonogenicity remained unchanged. These findings indicate the importance of age as a factor when designing cell-based therapies for older patients.

## Introduction

Mesenchymal stem/stromal cells (MSCs) hold promise in regenerative therapies due to their multipotency and availability. MSCs are being considered for the treatment of a wide range of pathologies, and researchers are especially interested in their potential to treat musculoskeletal disorders such as osteoarthritis, osteoporosis, and osteonecrosis [1]–[3]. Bone marrow is the most commonly investigated source tissue for these applications, although cells from other tissues like muscle and fat have also been used effectively [4]–[7]. Recent reports have determined that bone marrow-derived mesenchymal stem cells (BMSCs) can slow the degradation of articular cartilage or even regenerate it in osteoarthritic animal models [8], [9]. Similarly, muscle-derived stem cells (MDSCs) and adipose-derived stem cells (ASCs) have been used successfully in treating bone defects *in vivo* [10], [11].

The prevalence of the aforementioned musculoskeletal diseases in older populations has motivated researchers to investigate the impact of aging on MSC properties to evaluate their functionality for autologous treatments. Though conflicting results exist, past findings have indicated that the regenerative potential of MSCs deteriorates with age, which suggests a possible limitation in their use. In individual studies, old BMSCs and ASCs have diminished osteogenesis compared with their younger counterparts [12], [13]. Furthermore, aged MDSC proliferation is slowed compared with young MDSC rates [14]. Similar adverse aging effects have been reported for periosteal progenitor cells and even hematopoietic stem cells [15], [16]. While these past studies are beneficial for understanding how aging might affect a *single* MSC type, their experimental designs do not allow for a direct, comparative analysis of MSC types in a donor from multiple source tissues. Since MSC properties are variable among donors [17] and experimental approaches are often inconsistent, it is difficult to relate trends across literature to make a reliable and accurate conclusion. The effects of aging have not been compared among MSCs derived from different tissue sources of healthy donors, an important consideration since cell origin has also been shown to influence properties such as differentiation and proliferation [18]. Exploring whether tissue source disparately influences MSC susceptibility to adverse aging effects could identify a cell type that is minimally impaired, making this cell source an optimal candidate for use in regenerative therapies for older patients.

This study aimed to investigate the effects of aging on BMSCs, MDSCs, and ASCs derived in matched groups from young and old donor animals. We hypothesized that not all cell sources would see similar impairment of therapeutic characteristics with age. MSCs were isolated from rabbit donors, and their initial viabilities and yields were quantified to evaluate cell susceptibility to isolation procedures and determine the availability of cells in different tissue sources and age groups. Proliferation rates, cell senescence, and clonogenicity were quantified and compared among populations to examine the impact of aging on MSC expansion properties. Lastly, multilineage differentiation potential was investigated by quantifying the production of lineage-specific metabolites for adipogenesis, osteogenesis, and chondrogenesis.

## Materials and Methods

### MSC Isolation and Culture

BMSCs, MDSCs, and ASCs were isolated from young (4–6 months) and old (4–5 years), female, New Zealand white rabbits (RSI Farms, Mocksville, NC, USA) as previously described (N = 5 for each age group) [5], [19]–[21]. Brief summaries of these isolation procedures are provided below. To limit the effect of donor variability and strengthen comparisons among MSC sources, all three tissues were isolated from the same donor for each of the ten animals. The influence of isolating tissues from the same set of donors was investigated for all parameters listed below and included in the interpretation of the results. All animal work was performed in accordance with the local guidelines of the institutions and only after approval by the Institutional Animal Care and Use Committee (Permit numbers: 0015-10 and 0218-12) in accordance with the Guide for the Care and Use of Laboratory Animals published by the US National Institutes of Health (NIH Publication No. 85–23, revised 1996).

BMSCs were obtained from the long bones of individual rabbits following established protocols, with minor modifications [20]–[22]. Connective tissue was removed from femurs, which were then cut with bone scissors to expose the marrow cavity. Marrow was collected by centrifuging long bones at 400 g for 1 minute. Pellets were resuspended in BMSC culture medium (DMEM/F-12, 15% FBS, 1% antibiotic/antimycotic (Hyclone)), centrifuged, and then incubated in erythrocyte lysis buffer (155 mM NH_4_Cl, 10 mM K_2_CO_3_, and 0.1 mM EDTA) at room temperature for 10 minutes. The remaining cells were passed through a 70 µm strainer, centrifuged once more, and plated in BMSC culture medium (passage 0). Cells were counted before plating to determine viability and cell yield. To expand BMSCs, cells were maintained at 37°C, 5% CO_2_, and upon reaching 80% confluence, the cells were trypsinized and replated (passage 1). At passage 2, BMSCs were cryopreserved in freezing solution consisting of 80% FBS, 10% culture medium, and 10% dimethyl sulfoxide. Post-thawing, BMSCs were expanded once more (passage 3) before being used for experiments. This procedure (expansion to passage 3, post-thawing) was followed for all MSC types in this study.

MDSCs were isolated from hindlimb skeletal muscle using a modified preplate technique [5]. Briefly, muscle was washed with phosphate-buffered saline (PBS), and connective and fat tissues were removed. The remaining muscle tissue was minced and digested at 37°C in 0.2% type XI collagenase for one hour, then in 2.4 U/mL dispase for 45 minutes, and lastly, in 0.1% trypsin for 30 minutes. The resulting cell pellet was resuspended in MDSC culture medium (high glucose DMEM (DMEM/HG), 10% FBS, 10% horse serum (Hyclone), 0.5% chick embryo extract (United States Biological), and 1% antibiotic/antimycotic) and passed through a 70 µm strainer. Cells were then sequentially drawn through an 18G, 23G, and 27G needle, counted to determine cell viability and yield, and plated in collagen type I-coated flasks (Sigma). After two hours, the cell suspension was replated in new collagen-coated flasks. This process was repeated every 24 hours for an additional 3 days to isolate the slowly adhering cell fraction containing MDSCs. It should be noted that viability and cell yield were quantified immediately following cell isolation to be consistent with BMSC and ASC data sets. Actual MDSC yields within the harvest population could not be calculated since the cells were isolated only after the extended preplating procedure. To a certain extent, this is also true of BMSC and ASC harvests, which result in heterogeneous populations containing both stem and non-stem cells. Once preplating was completed, MDSCs were expanded in monolayer and cryopreserved at passage 2 as described above.

ASCs were isolated from inguinal fat pads using previously established methods [19]. Briefly, adipose tissue was washed thoroughly with PBS and then digested at 37°C for one hour in an equal volume of 0.1% type I collagenase. The sample was centrifuged and resuspended in ASC stromal medium (DMEM/F-12, 10% FBS, 1% antibiotic/antimycotic). After washing two more times with stromal medium, the cells were incubated in erythrocyte lysis buffer as described for BMSCs, passed through a 70 µm strainer, counted to quantify viability and yield, and plated in tissue culture-treated flasks. The following day, stromal medium was replaced with ASC culture medium consisting of DMEM/F-12, 10% FBS (Zen-Bio), 1% antibiotic/antimycotic, 0.25 ng/mL transforming growth factor-β1 (TGF-β1), 5 ng/mL epidermal growth factor, and 1 ng/mL fibroblast growth factor (R&D Systems) [19]. ASCs were expanded in monolayer to passage 2 and cryopreserved as described above.

### Population Doubling Time (PDT)

To examine the effects of aging on MSC proliferation, PDTs for each experimental group were quantified. Briefly, 2,500 cells were seeded in 6-well plates with their respective culture media (n = 24 for each experimental group). Cells from three wells per group were typsinized and counted every 1–2 days using a hemocytomer. Cell count data were plotted for each MSC population, and PDTs were calculated from the log phase of each growth curve [23].

### Colony Forming Unit (CFU) Assay

To assess how aging affects the clonogenicity of MSC populations, cells from each experimental group were plated at low seeding densities and allowed to form colonies. Based on previous empirical findings, 500 MDSCs, 1,000 ASCs, and 2,000 BMSCs were plated in 100 mm dishes in their respective culture media (n = 5). Following two weeks of growth, plates were fixed and stained with 0.5% crystal violet in methanol for 15 minutes. A dissection scope was used to count colonies comprising >50 cells and CFU formation was quantified by dividing the number of colonies by the number of cells originally plated. The CFU area was also determined by imaging plates with a Nikon D5000 digital camera (Nikon Corporation, Tokyo, Japan) and then processing with ImageJ software (National Institutes of Health).

### Senescence-Associated β-Galactosidase (SABG) Assay

MSC senescence was examined by staining for SABG [24], [25]. MSCs were plated at 5,000 cells/cm^2^ in 24 well plates (n = 3) and grown until ∼70% confluent. Monolayers were fixed with 0.5% glutaraldehyde and washed thoroughly with PBS. Plates were then stained with X-gal solution (1 mg/ml X-gal, 5 mM K_4_Fe(CN)_6_·H_2_O, 5 mM K_3_Fe(CN)_6_, 1 mM MgCl_2_ in PBS, pH 6) for 24 hours at 37°C. To visualize all cells, nuclei were labeled with 4′,6-diamino-2-phenylindole (DAPI, Thermo Fisher Scientific), and the number of cells positive for SABG activity versus total cells was quantified by brightfield and fluorescence imaging, respectively. For each well, at least 300 cells from 3–6 images were counted to obtain percentages.

### Multilineage Differentiation

#### Osteogenic Differentiation

steogenesis was assessed by quantifying alkaline phosphatase activity and calcified matrix deposition in response to chemical induction. MSCs were plated at 8,000 cells/well in 96-well plates with their respective culture media. Upon reaching confluence, monolayers were exposed to osteogenic (DMEM-HG, 50 ng/mL BMP-2 (a kind gift from Dr. Nic Leipzig), 10 mM β-glycerophosphate, 0.15 mM ascorbate-2-phosphate (Sigma), 1% antibiotic/antimycotic) or control (DMEM-HG, 10% FBS, 1% antibiotic/antimycotic) medium (n = 8 osteogenic, 8 control) [26]. Media were changed every 2–3 days, and after 1 week of induction, half of the wells (n = 4 osteogenic, 4 control) were assessed for alkaline phosphatase (ALP) activity using a BioVision ALP activity kit according to the manufacturer's protocol (Mountain View, CA). After a total of 3 weeks of induction, the remaining wells were fixed with 3.7% paraformaldehyde (Thermo Fisher Scientific), and alizarin red S (ARS, Sigma) stain was applied to visualize calcified matrix deposition for each sample. Following qualitative imaging, spectrophotometry was used to measure the optical density of eluted samples at 540 nm [27]. To compare osteogenesis between age groups and among tissue sources, the differentiation response of samples was quantified by normalizing ALP and ARS values of induced wells to their respective controls. To allow for analysis on a per-cell basis, nuclei stained with either Hoechst (Invitrogen) or DAPI were visually counted for each ALP and ARS sample prior to biochemical processing. Osteogenesis assays were only completed for MDSCs and BMSCs because ASC monolayers consistently became excessively contractile and balled-up during induction, preventing accurate assessment of matrix deposition and cell numbers.

#### Adipogenic Differentiation

Adipogenesis was assessed by quantifying intracellular lipid accumulation in response to chemical induction. MSCs were plated at 8,000 cells/well in 96-well plates with their respective culture media. Upon reaching confluence, monolayers were exposed to adipogenic (DMEM-HG, 10% FBS, 1.7 µM insulin, 1 µM dexamethasone, 0.2 mM indomethacin, 0.5 mM isobutyl-1-methylxanthine (Sigma), and 1% antibiotic/antimycotic) or control medium (n = 4 adipogenic, 4 control) [28]. Media were changed every 2–3 days for 2 weeks before fixing samples with 3.7% paraformaldehyde. Oil red O (ORO, Sigma) stain was then used to visualize intracellular lipid production. After qualitative imaging, ORO dye was eluted, and optical densities were measured at 500 nm using spectrophotometry [27]. To compare adipogenesis among MSC types, the differentiation response was quantified by normalizing optical density values of induced wells to their respective controls. Like with osteogenesis, DAPI-stained nuclei were counted for each sample to allow for analyses on a per-cell basis.

#### Chondrogenic Differentiation

Chondrogenesis was assessed by quantifying sulfated glycosaminoglycan (sGAG) synthesis in response to chemical induction. Cell pellets were formed by placing MSCs in V-bottom, 96-well plates at 50,000 cells/well and centrifuging for 5 minutes at 400 g. Culture medium was then replaced with either chondrogenic (DMEM-HG, 10 ng/ml TGF-β1, 0.15 mM ascorbate-2-phosphate, 100 nM dexamethasone, 1% ITS + Premix (BD Biosciences), and 1% antibiotic/antimycotic) or control medium (chondrogenic medium without TGF-β1) (n = 4 chondrogenic, 4 control) [19], [27], [29], [30]. Media were changed every 2–3 days for 3 weeks. For assessment, microtissues were digested with papain (Sigma), and sGAG amounts were quantified using the dimethylmethylene blue (DMMB) assay at pH 1.5 and measuring samples at 525 nm [27], [31]. To normalize sGAG amounts to approximate cell numbers, DNA content of samples was quantified using the PicoGreen assay (Invitrogen), with fluorescence measured at 480 nm excitation, 520 nm emission. Since some control samples produced no sGAGs, the differentiation response was quantified by normalizing induced values to the average of non-induced controls within each group.

### Statistical Analysis

Two-way ANOVA was used to detect differences across tissue sources and ages for initial cell yield and viability, proliferation, senescence, clonogenicity, and differentiation. Significance levels for individual comparisons were determined using a Tukey post-hoc test (p<0.05 for significance). Non-normal data sets were transformed prior to statistical analysis. Statistical comparisons of relative, metabolite production across tissues were not conducted since “optimal” differentiation cocktails for each individual MSC source could not be assured, making it inappropriate to directly compare across MSCs for this set of data. The differentiation frequency for each lineage was determined by quantifying the positive differentiation response between control and induced samples for each experimental group using a Student's t-test.

## Results

### Cell yields and viabilities following isolation

**Figure 1:**
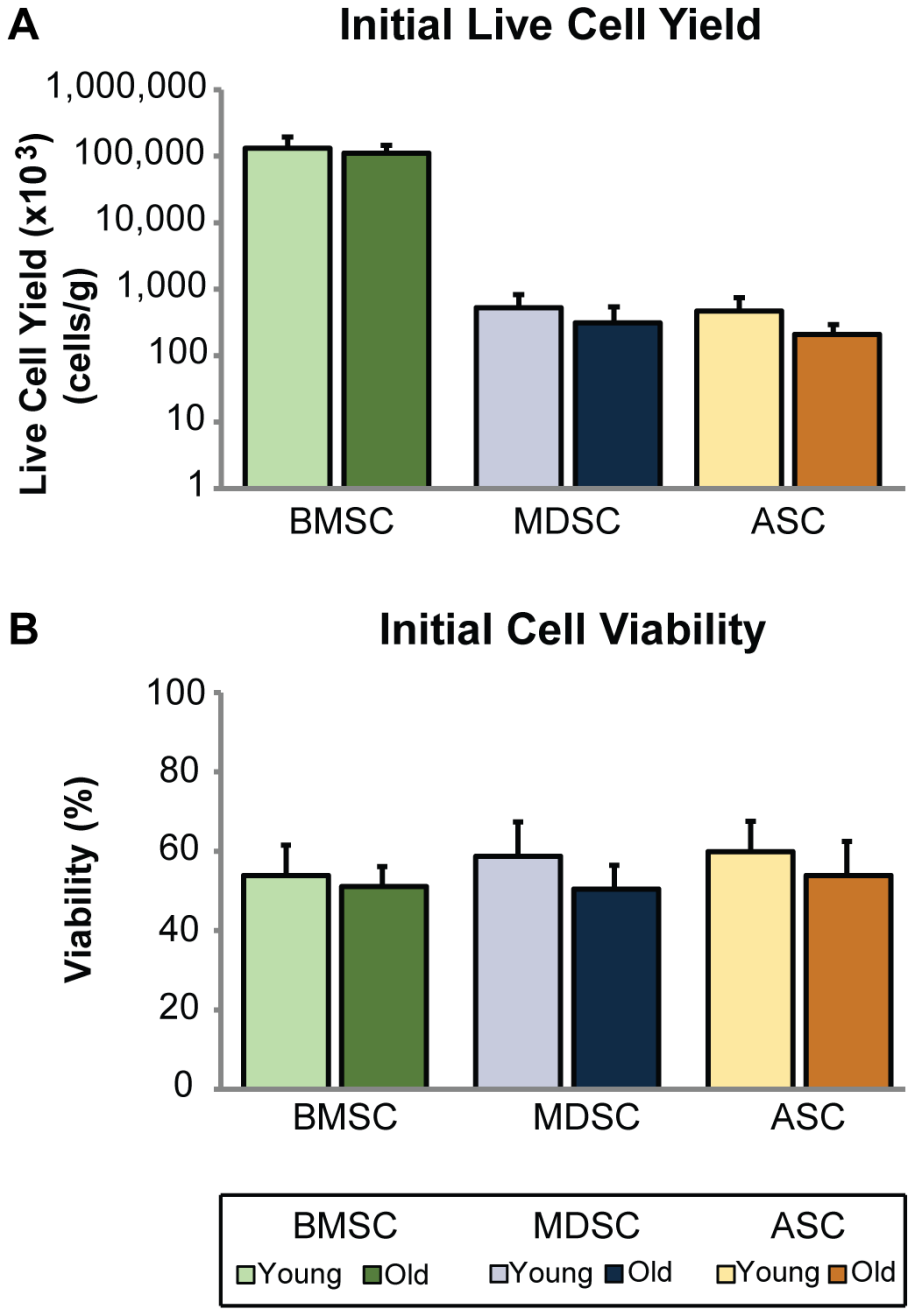
MSC yield and viability following isolation. Age adversely affected initial cell yield and viability of MSCs. After cell isolation, initial cell yield and viability were quantified. (A) Live cell counts were normalized to harvested tissue mass, with results indicating that cell yield was significantly reduced based on age as a factor (p<0.05). However, individual comparisons within tissue source showed no significant differences between age groups. BMSC yields were significantly larger than MDSC and ASC yields (p<0.001). (B) While viability was also reduced across MSC types, this trend did not quite reach significance (p = 0.051). Error bars depict standard deviations.

Cell yields and viabilities for freshly isolated young and old cell populations were quantified to examine how age influences cell availability within tissue sources as well as survival after isolation. For all tissue sources, old cell yields were significantly smaller than young by 15–55%, based on age as a factor (p<0.05, Fig. 1A). Cell yields ranged from 0.1–210×10^6^ cells/g, with BMSC yields being significantly greater than MDSC and ASC yields by at least 200-fold (p<0.001). As mentioned previously, yields reflect total cell numbers obtained from the tissue and not only MSCs. Although average, old cell viabilities for all tissue sources were 5–15% lower than young viabilities, this trend did not reach significance (p = 0.051, Fig. 1B). Overall cell viabilities were comparable among all three tissue sources, ranging from 42–67%, slightly lower than other reported findings, potentially due to differences in isolation procedures or animal model used [32]–[38].

### Expansion properties of young and old MSCs

**Figure 2:**
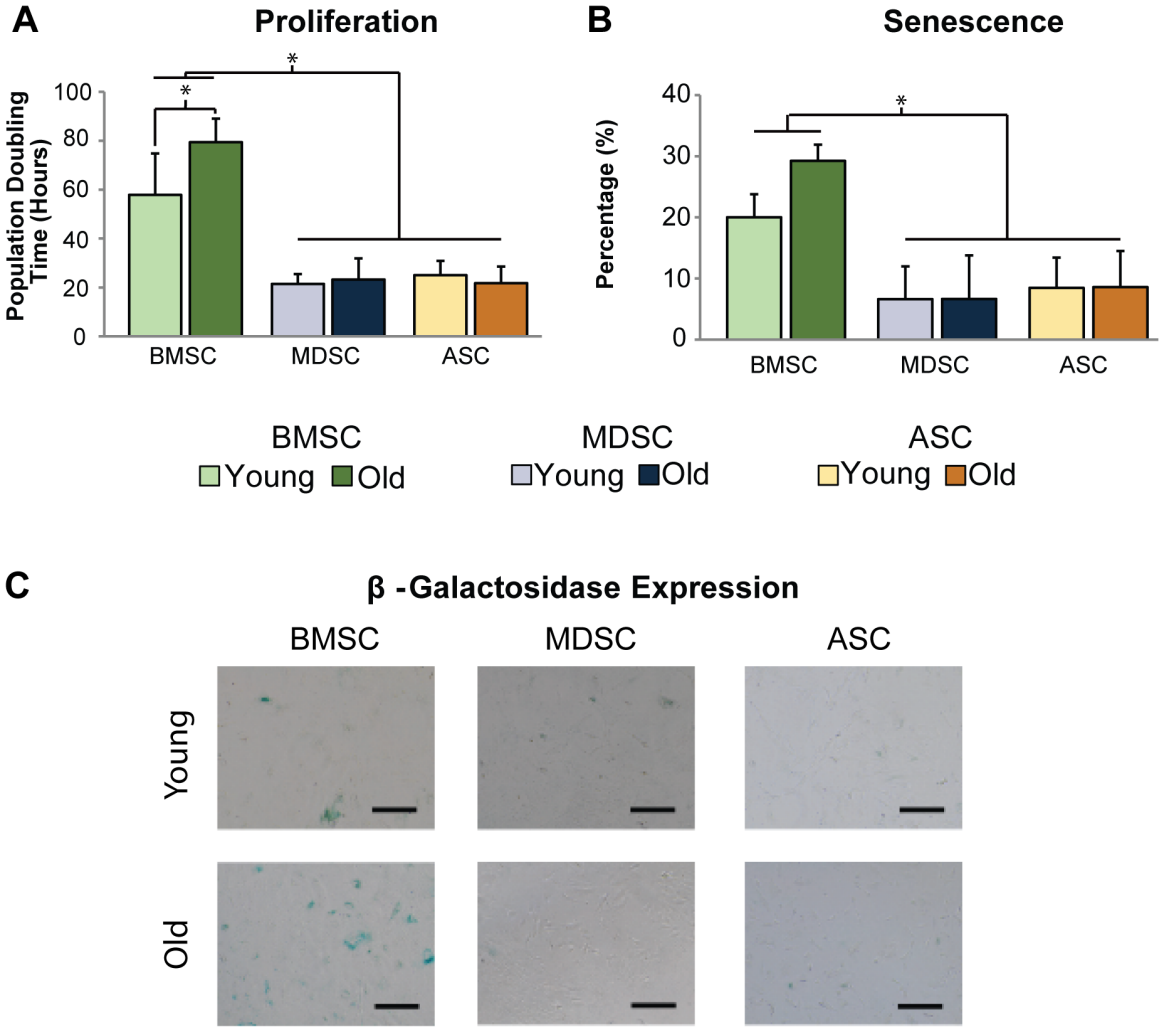
Cell expansion properties. Characterization of MSC expansion properties revealed differences in age-related changes across MSC populations. (A) PDTs of old BMSCs were significantly longer than young BMSC PDTs (p<0.005). However, no differences were observed between age groups for MDSCs or ASCs. Additionally, BMSC PDTs were significantly longer than MDSCs and ASCs (p<0.001). (B) Quantitative and (C) qualitative assessment of β-galactosidase staining showed that senescence was greater in old BMSC populations than young, although this trend did not reach significance (p = 0.095). MDSC and ASC senescence was not affected by age. Statistical analysis also determined that senescence was significantly greater in BMSC populations than MDSCs and ASCs (p<0.001). Error bars depict standard deviations. (Scale bars  = 100 µm).

To understand how MSC expansion properties are affected by age, PDT, SABG expression, and clonogenicity were examined for young and old cells. Quantifying the proliferation of MSCs during exponential growth revealed that BMSC PDT was impaired with donor age (36% slower, p<0.005), while PDTs for MDSCs and ASCs were not (p = 0.60, Fig. 2A). Comparisons across tissue sources revealed that BMSC population doubling times were significantly longer than MDSCs and ASCs on average by at least 130% (p<0.001).

Quantitative and qualitative assessment of X-gal staining showed that while SABG activity increased noticeably with donor age for BMSCs (46% higher), this trend did not reach statistical significance (p = 0.095, Fig. 2B, C). Conversely, no apparent differences were observed with age for MDSCs or ASCs (p = 0.88). Comparisons of senescence across tissue sources showed that BMSC SABG activity was at least 140% higher than in MDSC and ASC activity (p<0.001).

**Figure 3:**
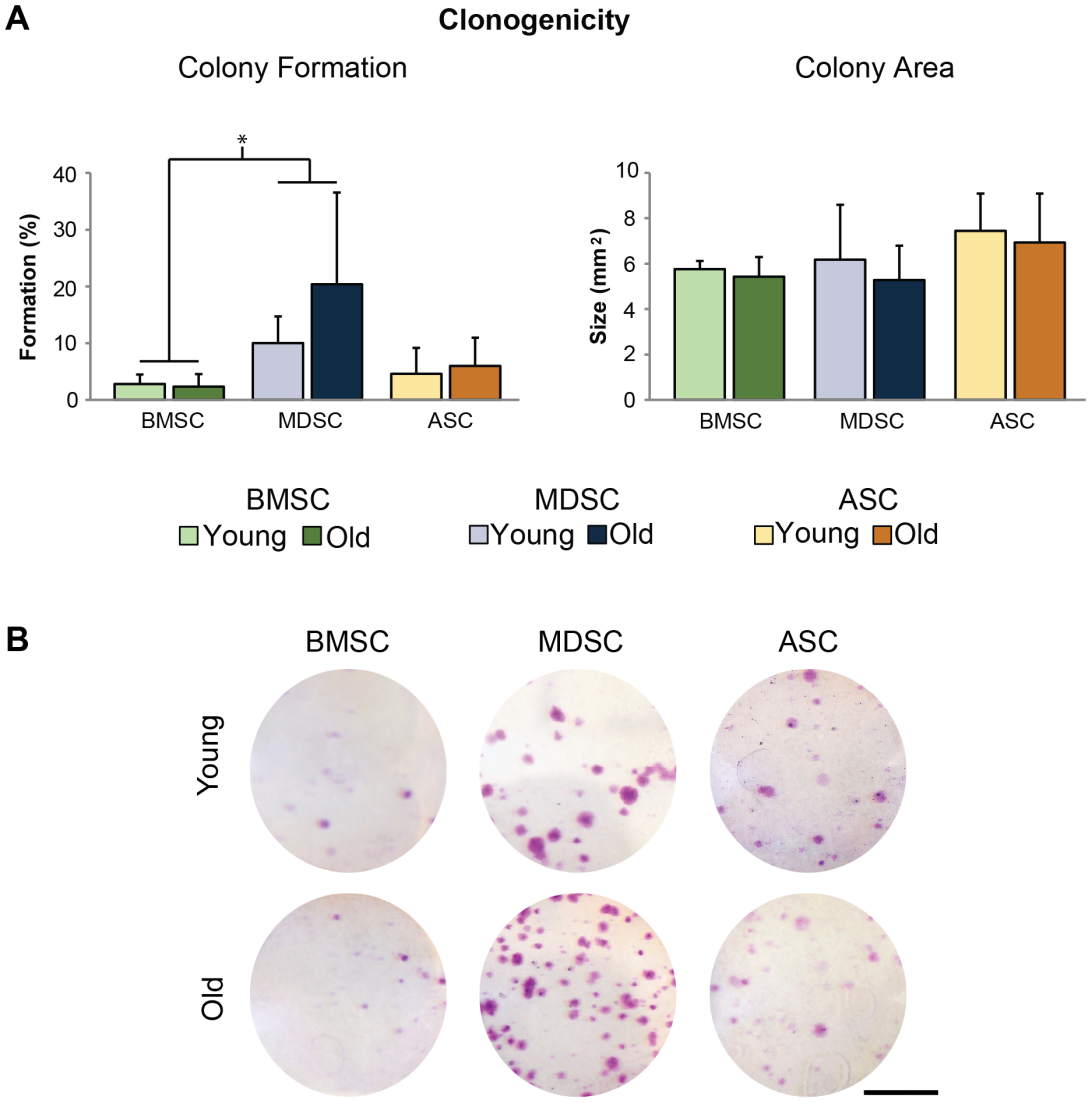
Clonogenicity of MSC populations. Quantification of colony formation and area indicated that age had no universal effect on MSCs for either parameter. However, MDSC colony formation was significantly higher than BMSC formation (p<0.005). Error bars depict standard deviations. (Scale bars  = 25 mm).

Clonal expansion of young and old MSC samples showed that CFU formation ranged from 0.4-40%, and were comparable between age groups for all MSC populations (p>0.25, Fig. 3A, B). Comparisons among tissue sources indicated that MDSC CFU efficiencies were 7-fold higher than BMSC efficiencies (p<0.005). ASC CFU efficiencies were not significantly different from BMSC (p = 0.22) or MDSC efficiencies (p = 0.051). Average CFU area ranged from 5.3–7.4 mm (Fig. 3A). However, we were unable to detect differences between the age groups for any MSC population (p>0.50) or tissue sources (p = 0.12).

### Multilineage differentiation potential of young and old MSCs

**Figure 4:**
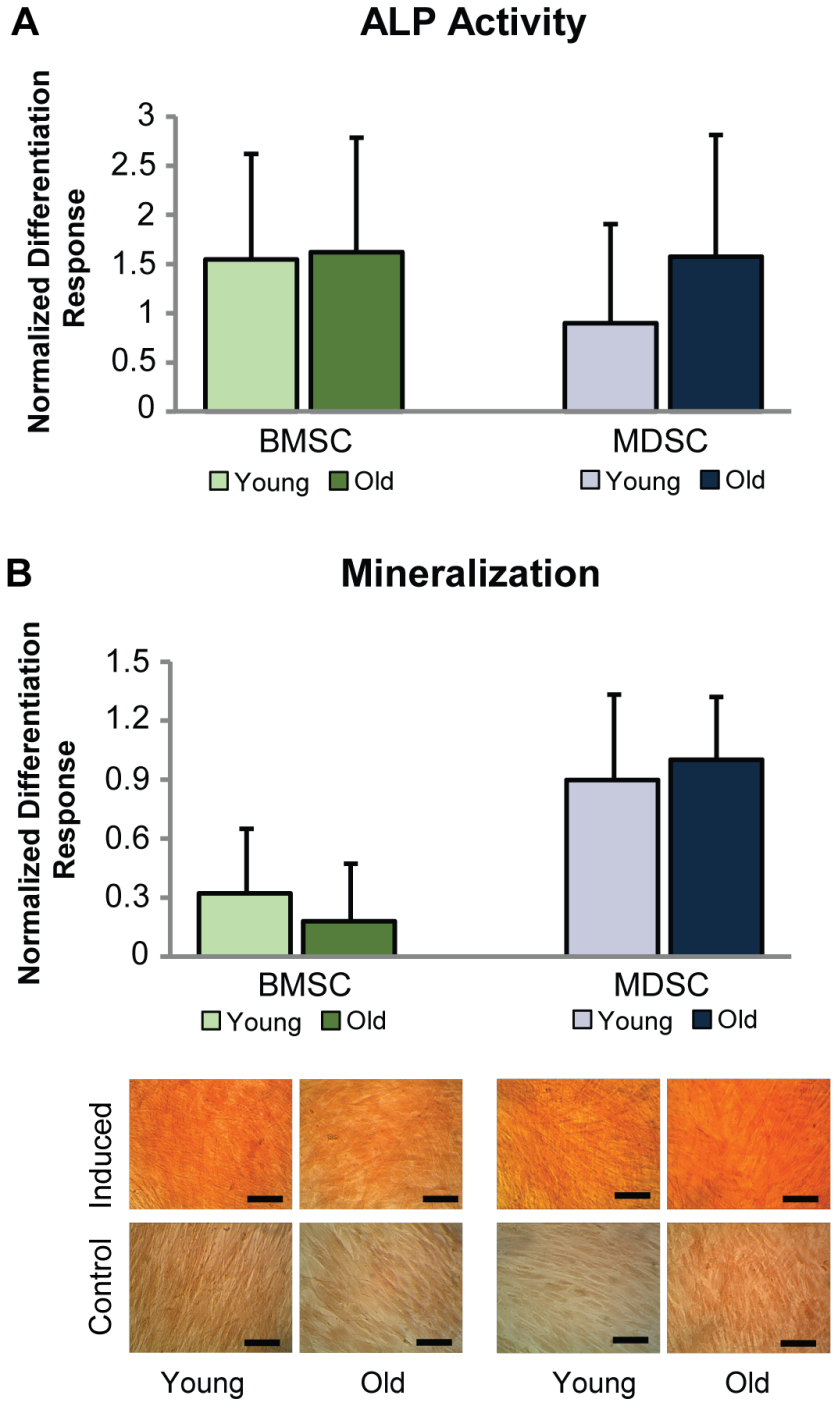
MSC osteogenic differentiation. Age did not adversely affect BMSC or MDSC osteogenesis. (A) Control-normalized differentiation response of ALP activity after 7 days of induction was comparable between age groups for BMSCs and MDSCs. (B) Calcified matrix production was examined quantitatively and qualitatively with ARS stain after 21 days of induction. Results were similar between age groups for BMSCs and MDSCs. ASC osteogenic differentiation was not characterized since cells became contractile upon induction and balled up, preventing accurate assessment. Error bars depict standard deviations. (Scale bars  = 100 µm).

Differentiation of young and old MSCs was evaluated based on lineage-specific metabolite production for the osteogenic, adipogenic, and chondrogenic lineages. Histological staining of osteogenic samples with ARS after 21 days of induction showed no differences in calcified matrix coverage between young and old cells within BMSC and MDSC groups (Fig. 4). Quantification of ALP activity on day 7 and calcified matrix deposition on day 21 showed that the normalized differentiation response was similar for young and old cells within BMSC and MDSC groups (p = 0.41, Fig. 4A, B). ALP and ARS raw values were also examined on a per-sample and per-cell basis, along with raw values for adipogenic and chondrogenic markers (Table 1). As mentioned previously, ASCs were not included in the osteogenic assessment due to complications caused by their excessively contractile phenotype.

**Table 1:** Detailed Differentiation Response of Young and Old MSCs.

			BMSC		MDSC		ASC	
Marker	Values	Differentiation Condition	Young	Old	Young	Old	Young	Old
ALP Activity^a^ (mU)	Raw (x10^-3^)	Induced Control	116±65 44±28	55±50 25±27	45±40 24±21	32±26 11±7	-	-
	Cell Numbers (x10^3^)	Induced Control	22±13 20±10	20±11 12±4	47±13 29±9	39±13 26±8	-	-
	Normalized (x10^-6^ per cell)	Induced Control	6.1±5.0 2.8±22	2.6±1.3 2.1±2.0	0.8±0.5 0.8±0.7	0.9±0.7 0.5±0.3	-	-
	Differentiation Frequency		4/5	4/5	2/5	3/5	-	-
Calcified Matrix^a^ (OD)	Raw (x10^-3^)	Induced Control	133±41 104±28	103±34 87±16	247±116 130±51	194±96 94±34	-	-
	Cell Numbers (x10^3^)	Induced Control	23±13 22±12	18±9 13±6	42±10 26±10	32±6 24±8	-	-
	Normalized (x10^-6^ per cell)	Induced Control	8.1±4.3 6.5±4.7	7.1±3.7 8.9±5.5	5.7±2.1 5.2±1.6	6.0±2.3 3.9±0.5	-	-
	Differentiation Frequency		3/5	2/5	5/5	5/5	-	-
Lipid^b^ (OD)	Raw (x10^-3^)	Induced Control	143±50 47±10	100±60 44±25	85±16 32±19	91±80 43±26	124±28 47±10	107±40 129±74
	Cell Numbers (x10^3^)	Induced Control	24±13 23±10	16±10 14±8	48±5 47±6	41±10 37±11	33±10 29±10	26±8 21±7
	Normalized (x10^-6^ per cell)	Induced Control	7.5±3.0 2.8±1.4	7.9±4.3 5.2±5.7	1.8±0.5 0.7±0.5	2.0±1.4 1.0±0.4	5.5±4.5 2.3±2.5	5.7±5.4 6.9±5.1
	Differentiation Frequency		5/5	3/5	5/5	4/5	4/5	1/5
sGAG^c^ (ug)	Raw	Induced Control	33.0±36.4 3.6±2.8	3.3±2.0 2.0±1.6	1.8±1.2 1.1±1.4	3.7±2.6 3.1±1.8	4.5±1.4 3.2±2.1	3.9±1.4 2.7±1.5
	DNA (ng)	Induced Control	127±86 171±97	45±12 86±62	42±20 96±54	35±9 77±28	39±25 80±20	36±20 70±23
	Normalized (x10^-3^ per ng DNA)	Induced Control	224±124 24±16	75±43 46±42	68±56 27±43	20±14 10±4	195±137 40±27	140±80 50±36
	Differentiation Frequency		5/5	2/5	2/5	2/5	4/5	4/5

Mean ± standard deviation is reported. No data were reported for ASC osteogenesis due to their contractile phenotype during differentiation. Differentiation frequency indicates number of donors successfully differentiating based on induced and control metabolite production. ^a^ Indicative of osteogenesis; ^b^ Indicative of adipogenesis; ^c^ Indicative of chondrogenesis.

**Figure 5:**
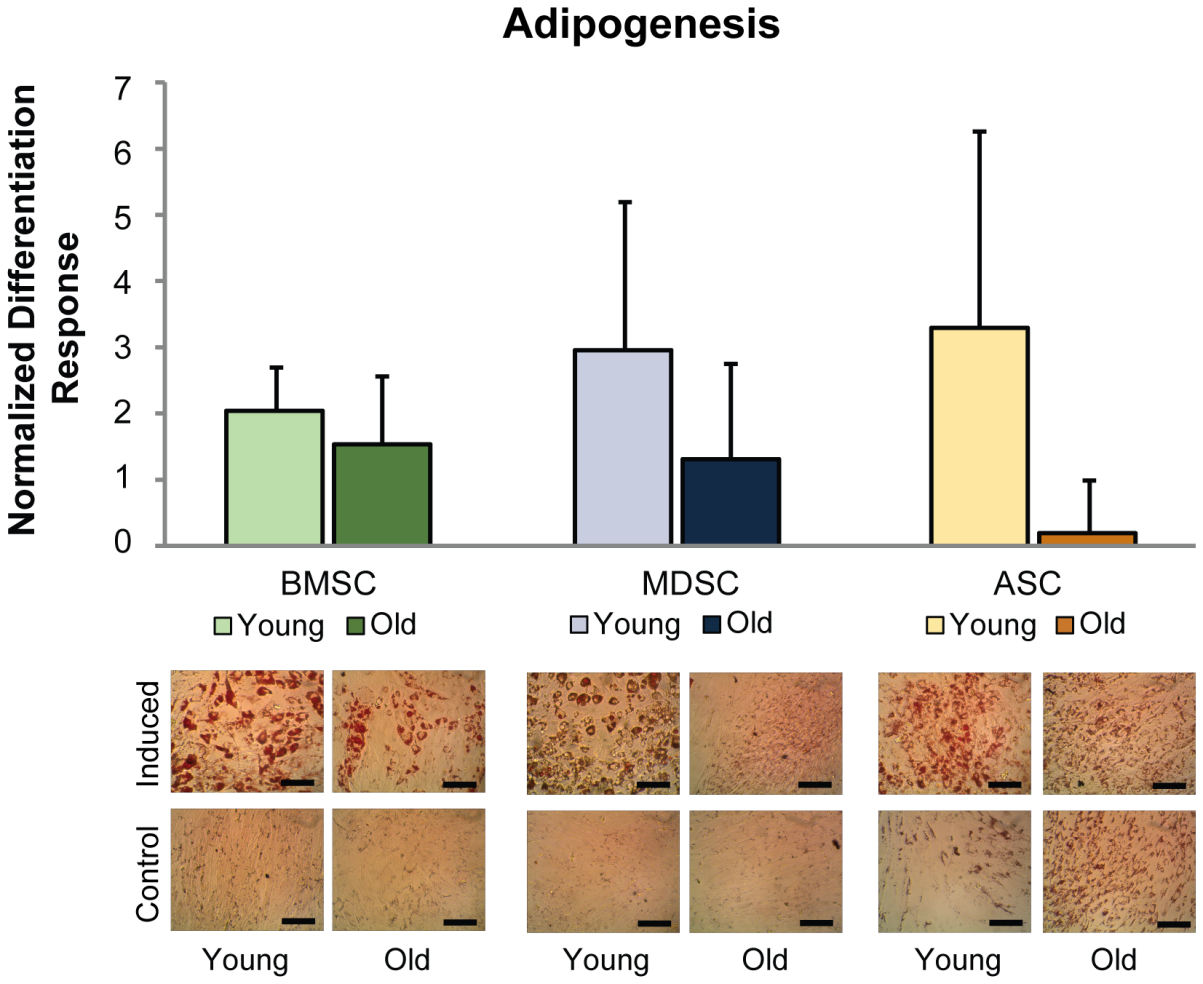
MSC adipogenic differentiation. Old age impaired the adipogenic potential of BMSCs, MDSCs, and ASCs. Quantification of eluted ORO stain revealed significant decreases in the overall, control-normalized differentiation response based on age as a factor (p<0.05). However, individual comparisons within tissue sources showed no significant differences between age groups. Qualitatively, staining showed that lipid production of induced samples was reduced for old BMSCs and MDSCs compared with their young counterparts. No readily apparent, age differences were observed for these cells in control conditions. Conversely, lipid production in old ASCs was noticeably greater than young ASCs in control conditions, while induced samples were similar. Error bars depict standard deviations. (Scale bars  = 100 µm).

Qualitative assessment of ORO staining revealed that chemically induced, old BMSC and MDSC populations produced less lipids than their respective young cell populations, whereas in control medium, no differences existed between young and old groups (Fig. 5). Interestingly, old ASCs in control medium exhibited more extensive lipid staining than young ASCs, but this difference was not observed for the induced condition. Quantifying ORO staining of young and old adipogenic cultures confirmed that this differentiation response was reduced with age for all MSC populations, based on age as a factor (p<0.05, Fig. 5). The most severe loss in adipogenic potential was observed for ASCs. Although lipid accumulated in old, induced ASC cultures, the normalized induction response was minimal since control samples also produced large amounts of lipids. Conversely, young ASCs differentiated as expected, with induced samples having more lipids than controls. Comparing between young and old cells revealed that the normalized differentiation response of old cells was only 6% that of young. Moreover, it was determined that raw adipogenic values of old ASCs were not significantly increased from controls (p = 0.27, Table 1) but were increased for young ASCs (p<0.01). For old BMSCs and MDSCs, the normalized differentiation response was 75% and 44% of young cells, respectively, but raw adipogenic values were still significantly increased compared to controls (p<0.05, Table 1).

**Figure 6:**
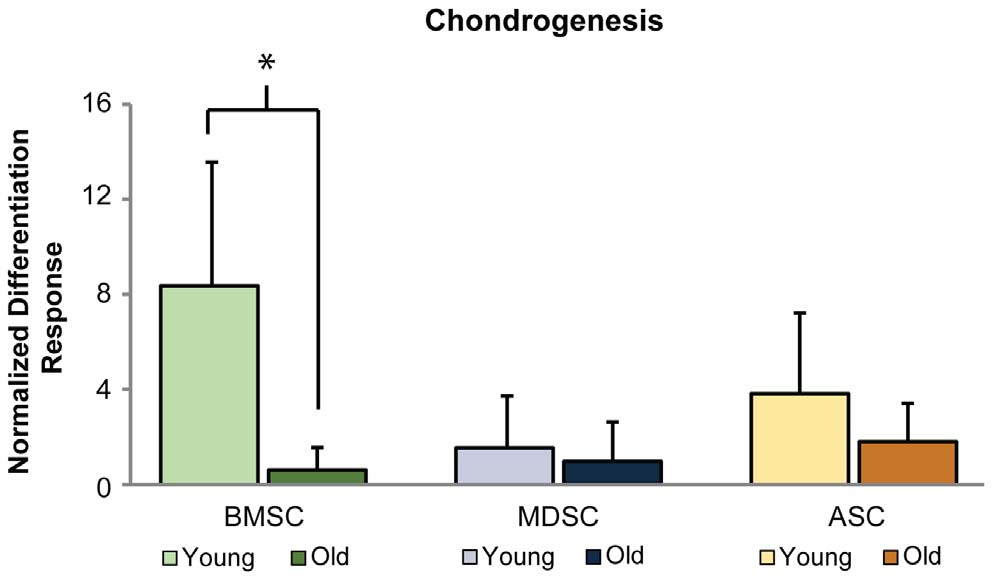
MSC chondrogenic differentiation. Chondrogenesis was reduced with age for BMSCs, but not for MDSCs or ASCs. Quantification of sGAG production showed that the control-normalized differentiation response of old BMSCs was significantly lower than young BMSCs (p<0.001). Conversely, no significant differences were observed between age groups for MDSCs or ASCs. Error bars depict standard deviations.

Chondrogenesis assessment showed that increases in sGAG production were significantly reduced with age for BMSCs (p>0.001) but not MDSCs or ASCs (p>0.33, Fig. 6). The normalized differentiation response of old BMSCs was only 7% of young BMSCs. Although BMSC chondrogenesis was reduced significantly with age, similar amounts of normalized and raw metabolite production were observed among all old MSC populations (Table 1). Quantifying the chondrogenic differentiation frequency of each MSC population showed that fewer old BMSC samples differentiated than young, while frequencies were comparable between age groups for MDSCs and ASCs (Table 1).

## Discussion

The results of this study indicate that donor age disparately affects tissue-specific MSC regenerative properties such as cellular proliferation, senescence, and differentiation potential. All MSC types showed lower cell yields and impaired adipogenesis with age. Old BMSCs exhibited slower PDTs, increased senescence, and inferior chondrogenesis, whereas old MDSCs and ASCs did not. Beyond these findings, very few differences were observed between the regenerative properties of young and old MSC cell types. For example, age had no effect on CFU formation or area measurements for any MSC population. Investigating the effects of age on MSC properties is a research area of high relevance due to the potential of MSCs for treating musculoskeletal disorders that are prevalent in older populations, including osteoarthritis and osteoporosis. Past studies conducted on individual MSC types have found that proliferation and differentiation potential are typically impaired with donor age [13], [14], [39]–[45], though this is not always the case. Few studies have investigated whether MSCs derived from different tissue sources are similarly susceptible to the aging process [46], and none have investigated this using healthy, donor-matched MSC comparisons. We hypothesized that since cell source has previously been shown to influence the abilities of MSCs to resist toxic environments like oxidative stress [47], specific tissues may also influence resident MSC resistance to adverse aging effects. Comparing how different tissue-derived MSCs are affected by aging can potentially identify an MSC type that best maintains its therapeutic potential in older populations, providing a preferred cell source for clinical procedures with these patients.

Differences in aging effects among MSC populations were observed for measured expansion properties. Results showing that age significantly affected PDT for BMSCs but not for MDSCs or ASCs suggest that BMSCs lose their proliferative properties earlier than MSCs from other tissues. Our data are consistent with previous studies investigating ASCs, BMSCs, or both in other experimental models, which found that aging slowed PDT in BMSCs but not ASCs [39], [40], [46], [48]–[50]. Few studies have examined the effects of age on MDSCs, but none have compared aging of MDSCs with other MSC populations [51]. Our data are the first to indicate that MDSCs are less susceptible to aging than BMSCs with respect to PDT. MDSCs and ASCs were also shown to be more resistant than BMSCs to age-related senescence, although this trend did not reach significance. An increase in senescence with age has been reported for MSCs from other species besides rabbit, including non-donor matched, human and rhesus macaque studies [46], [50]. An increase in senescent cells could account for the longer PDT measured for old BMSCs since fewer cells would be proliferating. The expansion properties of MSCs are important for their clinical use due to the large number of cells necessary for therapies. Longer PDTs and elevated senescence increase the amount of time that old BMSCs must be expanded for *in vitro*. Longer monolayer expansion times could potentially impair old BMSC differentiation potential when compared with young BMSCs [52], [53]. Use of MDSCs or ASCs may be preferable to BMSCs for regenerative therapies since their expansion properties are maintained with age.

Results showed that age had measurable, disparate effects on the *in vitro* differentiation response of BMSCs, MDSCs, and ASCs. This was primarily true for chondrogenesis, where there was a dramatic loss in BMSC chondrogenic potential with age but no effect for MDSCs or ASCs. While changes to BMSC chondrogenesis have been investigated previously, we found no studies that examined these effects for MDSCs or ASCs. Osteoarthritis is a prevalent pathology in older populations, and because there are currently no effective, long-term therapies, many MSC types are being considered as a treatment option. Since our results suggest that MDSCs and ASCs maintain their chondrogenic potential with age better than BMSCs, they may serve as promising candidates for cell-based cartilage therapies.

Lipid accumulation data revealed that age reduces the adipogenic differentiation response of BMSCs, MDSCs, and ASCs, which could be highly relevant for reconstructive and cosmetic surgery procedures. The most noticeable reduction in adipogenesis was for ASCs, where induced values were not significantly increased from controls. While these results suggest that old ASCs do not significantly respond to induction medium, large amounts of lipid were still produced in both induced and control samples. Therefore, old ASCs may still be viable for adipogenic regenerative therapies but limited since differentiation cannot be as well controlled through induction factors. Findings showed that old MDSCs and BMSCs still respond to adipogenic differentiation cocktails, but their application may be restricted since their differentiation response is reduced from that of young cells.

No aging effects were observed for BMSC or MDSC osteogenesis. ALP activity, an early osteogenic marker, and calcified matrix deposition, a late osteogenic marker, were similar for young and old BMSCs and MDSCs. These data suggest that the osteogenic differentiation potential of BMSCs and MDSCs is not affected by age and that both MSC sources could be considered for bone-related applications. Our findings related to BMSC osteogenic potential are supported by other groups, emphasizing the importance of these cells for treating bone damage in elderly populations [54], [55]. However, other factors such as decreased BMSC numbers and slowed proliferation rates should also be considered. MDSC osteogenic potential has not been thoroughly investigated, and these are the first data to determine that MDSC osteogenesis is maintained with age. The ability of both young and old MDSCs to undergo osteogenesis supports their use in regenerative treatments.

Age reduced overall cell yield for populations isolated from bone marrow, muscle, and adipose tissue, consistent with previous reports [13], [56]. And while not statistically significant, initial cell viability was also reduced with age for all MSC populations. The effects of aging on harvested cell viability are rarely reported, but this is a critically important parameter for determining how many usable cells are actually available for a treatment. Having a reduction in cell yield and viability is concerning since it suggests that many older tissue sources might not provide a large abundance of cells for regenerative medicine applications. The reduction in initial viability with age might indicate why there was a decrease in initial cell yield in older populations. If more cells are undergoing apoptosis or necrosis in old tissues, or are too fragile to withstand isolation procedures, fewer cells are available for therapeutic applications.

While our results show that age reduces the differentiation response of some MSC populations, this reduction does not necessarily indicate that they have less regenerative potential than “unimpaired” MSCs. For example, BMSC chondrogenesis was reduced with age while MDSC and ASC chondrogenesis was maintained. However, when comparing total sGAG/DNA values, chondrogenesis for old BMSCs was still much more robust than for old MDSCs, even after undergoing age-related reductions in differentiation potential. For many applications, the absolute amount of matrix production is what matters, regardless of how much it has decreased from peak levels. Ultimately, *in vivo* studies will be necessary to compare actual therapeutic potentials for each MSC type, taking into account total matrix production as well as other important characteristics such as cell yield, viability, and proliferation. While the reported differentiation potential, expansion properties, and cell yield data are consistent with current literature, some discrepancies do exist. Dissimilarities in these trends could be due to many factors, including donor variability, or differences in animal model systems, age groups, and culture conditions. Contrasting isolation methods could also contribute to disparities. One notable result that differed from other studies is that we observed no reduction in BMSC osteogenesis with age. Our observations could be influenced by the age range assessed. It is possible that the osteogenic potential of rabbit BMSCs is impaired at a younger age than even the “young” group examined in this study. A dramatic decrease in osteogenesis at early ages has been reported previously [50], [57]. Examining neonatal rabbit BMSC osteogenesis would confirm whether differentiation potential is reduced prior to 4–6 months of age. Other discrepancies could also be explained by the different model systems used in various studies. For example, the significant reduction in rabbit ASC and BMSC adipogenesis we observed is consistent with multiple, mouse MSC studies [44], [58]. However, other groups studying human BMSCs or ASCs reported no age-related reduction in adipogenesis [49], [59], suggesting that some results may be specific to smaller animal models. Discrepancies could also be attributed to donor-to-donor variability and differences in methodology, since results have been shown to vary widely within a single species [6], [46], [56], [58]. The number of contradicting reports suggests that comparisons across studies of single MSC types is not reliable and stresses the need to directly compare aging effects among MSC sources within individual experiments, as was done in this study. Isolating MSCs from a single donor is likewise important to help minimize the effects of species and donor-to-donor variability.

The cell populations examined in this study are heterogeneous in their composition, consisting of stem and progenitor cells as well as fully differentiated cell types. While antigen-based purification was not used, empirical results for multipotency and clonogenicity suggest that MSCs comprised a large proportion of these populations. Furthermore, surface marker profiles can be inconsistent across literature [60]–[62], and “purified” MSCs are still inherently heterogeneous, with variable regenerative potential [29]. Many studies have investigated MSCs without purification [49], [58], [59], and evidence exists that separating these cells into antigen-specific populations may even impair their differentiation response compared to the unsorted cells [63]. Clinical applications reflect this in the use of whole bone marrow versus purified BMSCs in many transplantation procedures [64], making the assessment of non-purified MSC populations equally as important as purified populations.

The current study used a rabbit animal model to allow for isolation of multiple MSC types in sufficient numbers from young and old donors. This experimental design is not feasible with smaller animals, particularly since fat tissue, and the ASCs residing in it, does not exist in sufficient amounts in rat and mouse pups. Investigating young and old age groups is complicated for longer-lived, large animal models, leaving rabbits as the most viable option. While the observed results may not be completely conserved across other experimental models, rabbits have been used extensively and reliably in multiple MSC studies focusing on regeneration of age-related musculoskeletal disorders [8], [21], [65]. This suggests that the findings and conclusions presented in this work should be applicable to future studies regardless of model system. Furthermore, rabbits were an appropriate model to isolate bone marrow, muscle, and adipose tissue from the same donors, a procedure that would be much less feasible in humans. For most parameters and MSC types, donor-dependent trends were not present (S1 Fig.). In other words, a specific donor could not be categorized as “strongly” or “weakly” regenerative, with all MSCs performing similarly well or poor in comparison with other donors. Rather, the relative ranking of MSC performance *within* a donor was variable, lending support to having an experimental design that incorporates matched-donor MSCs. For the current study, comparisons among the MSC types are more reliable than if each cell type came from a separate donor animal, which would simply exacerbate the effects of donor-to-donor variability.

## Conclusion

Understanding the effects of age on MSC properties is important due to their potential use in treating musculoskeletal disorders that are prevalent in older populations. Furthermore, comparing the effects of aging on different MSC populations could help optimize treatments by identifying an MSC source that is not adversely affected by age. This study compared the regenerative properties of young and old BMSCs, MDSCs, and ASCs. We determined that aging effects on MSCs from different source tissues were dissimilar. In particular, old BMSCs suffered from reduced chondrogenic potential and impaired expansion properties, while MDSCs and ASCs did not. Additionally, the adipogenic potential of all MSC types was adversely affected by aging. Although aging adversely affected the properties of some MSCs more than others, total metabolite production was still comparable across all three MSC types. Keeping in mind the aforementioned caveats regarding stem cell-specific induction cocktails, these results suggest that old BMSCs, MDSCS, and ASCs are equally promising candidates for therapies targeting older populations. Further investigation into the mechanism responsible for differential changes between young and old cells among MSC sources is warranted and, along with the presented findings, can be used to inform future studies focused on designing musculoskeletal treatments for the world's rapidly aging population.

## Supporting Information

S1 Fig **Influence of using matched-donors.** Isolating MSC populations from the same donor rabbits enabled us to examine patterns in cell properties both within and across donors. A graph of chondrogenesis reveals that trends among BMSCs, MDSCs, and ASCs are inconsistent within each donor. No evidence supported the existence of uniformly “strong” or “weak” donors, with some MSC types performing well for one donor but not for others. Donor-to-donor variability was still high, but by isolating all three cell types from single animals, the impact of this variability was lessened, increasing overall confidence in the study's conclusions. Error bars depict standard deviations.
(TIF)
