# Supplementary figures and images for: Impact of Aging on the Regenerative Properties of Bone Marrow-, Muscle-, and Adipose-Derived Mesenchymal Stem/Stromal Cells
Source: PLoS One. 2014 Dec 26;9(12):e115963. doi: 10.1371/journal.pone.0115963 (PMC4277426; doi:10.1371/journal.pone.0115963)

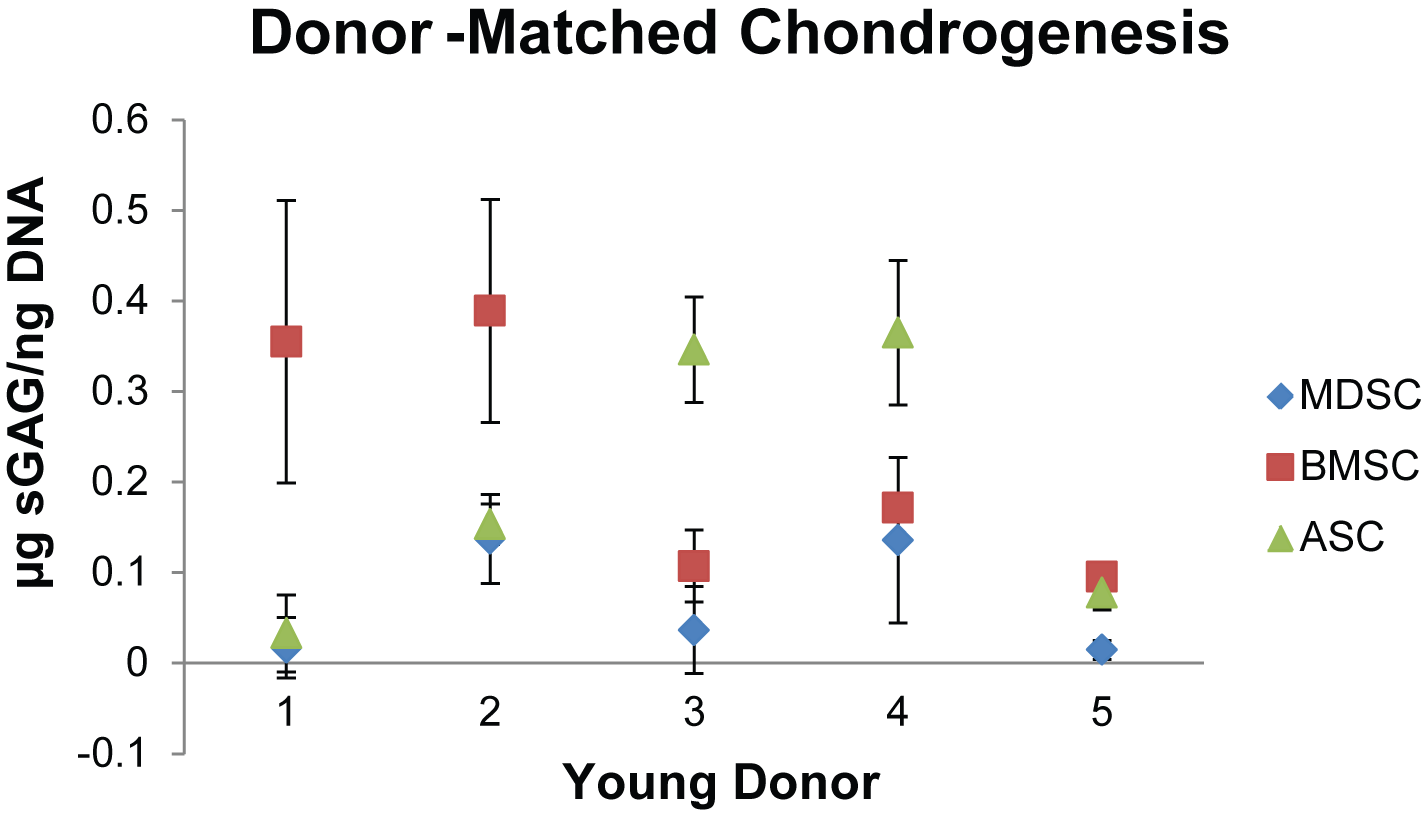

Supplement: S1 Fig — Influence of using matched-donors. Isolating MSC populations from the same donor rabbits enabled us to examine patterns in cell properties both within and across donors. A graph of chondrogenesis reveals that trends among BMSCs, MDSCs, and ASCs are inconsistent within each donor. No evidence supported the existence of uniformly “strong” or “weak” donors, with some MSC types performing well for one donor but not for others. Donor-to-donor variability was still high, but by isolating all three cell types from single animals, the impact of this variability was lessened, increasing overall confidence in the study's conclusions. Error bars depict standard deviations. (TIF) [file pone.0115963.s001.tif]
